# Effects of Levocarnitine on Brachial-Ankle Pulse Wave Velocity in Hemodialysis Patients: A Randomized Controlled Trial

**DOI:** 10.3390/nu6125992

**Published:** 2014-12-22

**Authors:** Terumi Higuchi, Masanori Abe, Toshio Yamazaki, Mari Mizuno, Erina Okawa, Hideyuki Ando, Osamu Oikawa, Kazuyoshi Okada, Fumito Kikuchi, Masayoshi Soma

**Affiliations:** 1 Department of Nephrology, Keiai Hospital, Tokyo 173-0036, Japan; E-Mails: thiguchi@keiai-hospital.jp (T.H.); zakio@keiai-hospital.jp (T.Y.); mari@keiai-hospital.jp (M.M.); erina@keiai-hospital.jp (E.O.); 2 Division of Nephrology, Hypertension and Endocrinology, Department of Internal Medicine, Nihon University School of Medicine, 30-1 Oyaguchi Kami-cho, Itabashi-ku, Tokyo 173-8610, Japan; E-Mails: oikawa.osamu@nihon-u.ac.jp (O.O.); kokada@med.nihon-u.ac.jp (K.O.); souma.masayoshi@nihon-u.ac.jp (M.S.); 3 Department of Cardiology, Keiai Hospital, Tokyo 173-0036, Japan; E-Mail: ando@keiai-hospital.jp; 4 Department of Nephrology, Meirikai Chuo General Hospital, Tokyo 114-0001, Japan; E-Mail: fkikuchi@ims.or.jp

**Keywords:** atherosclerosis, brachial-ankle pulse wave velocity, carnitine, end-stage kidney disease, hemodialysis

## Abstract

Background and Aims: Atherosclerotic cardiovascular disease is the most common cause of mortality in patients with end-stage kidney disease. Chronic kidney disease patients often exhibit a deficiency in l-carnitine due to loss during hemodialysis (HD). We studied the effects of l-carnitine supplementation on brachial-ankle pulse wave velocity (baPWV), a marker of atherosclerosis, in HD patients. Methods: This was a prospective, open-label, randomized, parallel controlled, multi-center trial testing the anti-atherosclerotic efficacy of oral l-carnitine administration (20 mg/kg/day). HD patients (*n* = 176, mean age, 67.2 ± 10.3 years old; mean duration of HD, 54 ± 51 months) with plasma free l-carnitine deficiency (<40 μmol/L) were randomly assigned to the oral l-carnitine group (*n* = 88) or control group (*n* = 88) and monitored during 12 months of treatment. Results: There were no significant differences in baseline clinical variables between the l-carnitine and control groups. l-carnitine supplementation for 12 months significantly increased total, free, and acyl carnitine levels, and reduced the acyl/free carnitine ratio. The baPWV value decreased from 2085 ± 478 cm/s at baseline to 1972 ± 440 cm/s after six months (*p* < 0.05) to 1933 ± 363 cm/s after 12 months (*p* < 0.001) of l-carnitine administration, while no significant changes in baPWV were observed in the control group. Baseline baPWV was the only factor significantly correlated with the decrease in baPWV. Conclusions: l-carnitine supplementation significantly reduced baPWV in HD patients. l-carnitine may be a novel therapeutic strategy for preventing the progression of atherosclerotic cardiovascular disease.

## 1. Introduction

Dialysis-related carnitine deficiency is characterized by decreased circulating free carnitine and increased levels of the acylated form. Free carnitine levels decrease principally as a result of dialytic loss, whereas decreased renal clearance and impaired β-oxidation of fatty acids lead to the accumulation of acylcarnitine and an abnormally high ratio of plasma acylcarnitine to free carnitine [1,2,3]. The primary known functions of carnitine are to transport long chain fatty acids into the mitochondria for β-oxidation and to completely remove metabolized acyl CoA. Thus, carnitine deficiency can result in impaired mitochondrial β-oxidation, decreased glucose oxidation, and ultimately in accelerated cellular apoptosis [4,5]. In addition, incompletely metabolized fatty acids associated with dialysis-related carnitine deficiency may interfere with metabolic pathways and contribute to the activation of inflammatory cascades. In turn, these abnormalities may contribute to clinical disorders, including cachexia, dyslipidemia, erythropoiesis-stimulating agent (ESA)-resistant anemia, insulin resistance and glucose intolerance, muscle weakness, and myopathy, and to intradialytic symptoms such as muscle cramps, hypotension, and cardiac arrhythmia [6,7]. This constellation of abnormalities resembles malnutrition-inflammation complex syndrome (MICS) or malnutrition–inflammation–atherosclerosis (MIA) syndrome, a strong predictor of outcome in end-stage kidney disease (ESKD) [8,9].

Classical risk factors for cardiovascular disease (CVD), such as dyslipidemia, hypertension, and diabetes mellitus, are prevalent in hemodialysis (HD) patients, but cannot explain the high frequency of CVD [10]. Alternatively, several recent studies have suggested that inflammation and oxidative stress may also contribute to the development of atherosclerotic CVD [11,12,13], and l-carnitine therapy can decrease markers of oxidative stress and inflammation [14,15,16,17]. Thus, l-carnitine may mitigate both the immediate cellular effects of dialysis-related carnitine deficiency and systemic processes underlying CVD. However, there are few studies examining whether l-carnitine supplementation can improve atherosclerosis. The present study was designed to investigate the effects of prolonged (12-month) l-carnitine supplementation on brachial-ankle pulse wave velocity (baPWV), a marker of atherosclerosis, in HD patients.

## 2. Methods

### 2.1. Patients and Study Protocol

This prospective, open-label, randomized, parallel controlled, multi-center trial examined 176 HD patients (mean age, 67.2 ± 10.3 years old; mean duration of HD, 54 ± 51 months) randomly assigned to an oral l-carnitine therapy group (*n* = 88) or a control group (*n* = 88). Patients were monitored for 12 months during control or l-carnitine treatment (administered at 20 mg/kg/day). Subjects were randomly assigned to two groups prior to the start of the study. An independent investigator with no previous knowledge of the subjects before commencement of the trial monitored randomization of subject entry order. Dynamic balancing randomization was carried out based on age, gender, HD duration, hemoglobin level, and presence or absence of diabetes mellitus. Thus, we ensured that there were no significant differences in baseline characteristics between the groups. The details of the assignment were then given to four independent investigators. The study protocol was approved by the Ethics Committee of Keiai Hospital and all patients gave written informed consent (Clinical Trial Registration number: UMIN000015185). The study protocol was designed in accordance with the Declaration of Helsinki. All patients were treated with HD therapy three times weekly in 4-h sessions at the blood purification unit of our hospital.

Enrollment criteria for were (1) age ≥20 years or ≤85 years, (2) ankle-brachial systolic pressure index (ABI) ≥ 0.9 or ≤ 1.4, and (3) plasma free carnitine concentration <40 μmol/L. Exclusion criteria were as follows: (1) age of <20 years or >85 years; (2) ABI of <0.9 or >1.4; (3) a history of amputation of extremities or the presence of peripheral arterial disease; (4) a history of severe heart failure, angina, myocardial infarction, or stroke within the past six months; (5) the presence of infectious disease, thyroid disease, malignant tumors, or treatment with steroids or immunosuppressants; (6) currently hospitalized; (7) poor oral food intake; and/or (8) l-carnitine supplementation within the past six months. Patients continued their regular medications, such as anti-hypertensives, ESAs, phosphate binders, and lipid-lowering agents, during the study period. Patients were regularly given dietary guidance, especially those under dietary restrictions (such as for salt and protein intake).

### 2.2. Study Evaluations

Blood cell counts, total bilirubin, aspartate aminotransferase, alanine aminotransferase, alkaline phosphatase, serum creatinine, blood urea nitrogen, electrolytes, uric acid, serum iron, total iron binding capacity, serum ferritin, total cholesterol, low-density lipoprotein cholesterol, high-density lipoprotein cholesterol, triglyceride, total protein, and albumin were measured by routine clinical chemistry procedures using commercial kits. High-sensitivity C-reactive protein (hs-CRP) and serum β2-microglobulin were measured by latex agglutination. Intact parathyroid hormone was measured by radioimmunoassay. Serum carnitine levels were determined by enzyme cycling methods as described previously [18]. Predialysis systolic and diastolic blood pressure values were recorded monthly. Blood samples were obtained before the start of an HD session. The baPWV and ABI were measured by “form PWV/ABI^®^” (OMRON COLIN Co., Ltd., Tokyo, Japan) at baseline and after six months and 12 months of oral l-carnitine treatment (or equivalent times for the control group) before weekly HD sessions on Wednesday or Thursday. The change in baPWV (ΔbaPWV) was defined as the difference between the baPWV value at baseline and that at the end of the study. Erythropoietin responsiveness index (ERI) was defined as the average weekly ESA dose divided by clinical dry weight and average blood hemoglobin (weekly ESA dose (units)/dry weight (kg)/hemoglobin (g/dL)) as described previously [19] to normalize the amount of required ESA to the severity of anemia. Other variables were evaluated at baseline and after 12 months of drug treatment.

### 2.3. HD Procedure

In all patients, the HD procedure was performed using dialyzers containing high-flux membranes (such as polysulfone, polyester-polymer alloy or cellulose triacetate) at a blood flow rate of 200–250 mL/min and a dialysate flow rate of 500 mL/min. The surface area of the dialyzer membrane was selected according to patient body weight. The glucose concentration of the dialysate was 100 mg/dL. Heparin was administered at 2600–5000 U per 4-h HD session for anticoagulation. The volume of ultrafiltration was maintained on the basis of clinical dry weight during each session.

### 2.4. Statistical Analysis

Results are expressed as the mean ± SD. We assessed differences between baseline and 12-month values using the *t*-test for paired data. The unpaired *t*-test was used to compare baseline means between treatment groups. Differences in post-treatment means were evaluated using the unpaired Student’s *t*-test for parametric data and the Mann-Whitney test for non-parametric data. Categorical data were compared using repeated-measures analysis of variance. Pearson’s correlation coefficients were computed to evaluate the relationship between ΔbaPWV and other measured parameters. Multiple stepwise regression analysis was performed to identify independent correlates of ΔbaPWV. SPSS ver. 20 (Chicago, IL, USA) was used for all analyses. Statistical significance was established at *p* < 0.05.

## 3. Results

### 3.1. Baseline Demographic and Clinical Data

**Figure 1:**
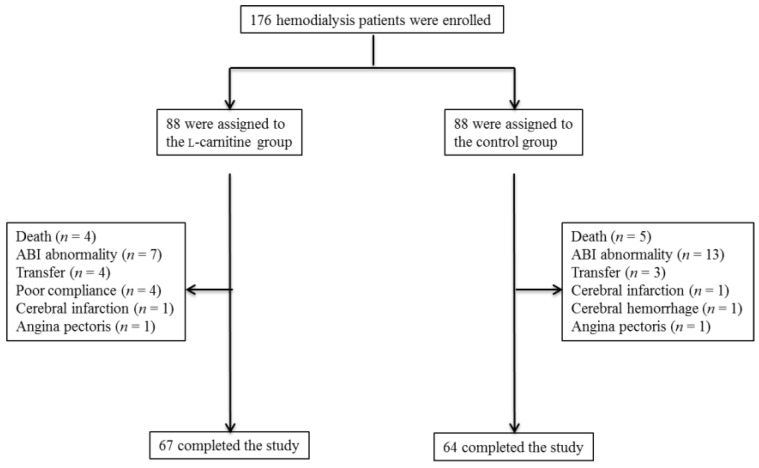
Enrollment, randomization, and monitoring of the study patients. ABI, ankle-brachial systolic pressure index.

One hundred seventy-six HD patients were enrolled in this trial and randomly allocated to the l-carnitine treatment group or the control group (88 patients per group). There were no significant differences in baseline demographic, metabolic, hemodynamic, anthropometric, or inflammatory variables between groups (Table 1). Forty-four patients did not complete the assessment or treatment, 21 in the l-carnitine group (23.8%) and 24 in the control group (27.2%). The other 67 patients in the l-carnitine group and 64 in the control group were included in the final analysis (Figure 1). In the l-carnitine group, subjects were excluded from the final analysis due to ABI abnormality (*n* = 7); poor adherence (*n* = 4); transfer to other hospitals (*n* = 4); hospitalization due to cerebral infarction (*n* = 1); angina pectoris (*n* = 1); or death from CVD (*n* = 2) or sepsis (*n* = 2). In the control group, exclusions were due to ABI abnormality (*n* = 13), transfer to other hospitals (*n* = 3), hospitalization due to cerebral infarction (*n* = 1), cerebral hemorrhage (*n* = 1), angina pectoris (*n* = 1), or death from CVD (*n* = 4) or sepsis (*n* = 1). During the study period, angiotensin receptor blocker (ARB) treatment was interrupted in one patient and the calcium channel blocker (CCB) dose reduced in three other patients of the l-carnitine group. In the control group, ARB treatment was initiated in two patients and the CCB dose was increased in one patient and reduced in two others.

**Table 1:** Baseline demographic and clinical variables.

	l-carnitine Group (*n* = 88)	Control Group (*n* = 88)	*p* Value
Male/female	63/25	65/23	NS
Age (years)	67 ± 11	68 ± 10	NS
Duration of dialysis (months)	54 ± 50	52 ± 50	NS
Diabetes mellitus (%)	54.5	52.3	NS
Smoker (%)	15.9	13.6	NS
Medication
Antihypertensive agents (%)	75	77.2	NS
Statins (%)	35.2	37.5	NS
Kt/V	1.32 ± 0.16	1.32 ± 0.17	NS
Hemoglobin (g/dL)	11.0 ± 0.9	11.1 ± 1.0	NS
BUN (mg/dL)	56.3 ± 13.6	57.7 ± 10.7	NS
Creatinine (mg/dL)	9.57 ± 2.93	9.21 ± 2.20	NS
Uric acid (mg/dL)	6.9 ± 1.1	6.7 ± 0.8	NS
Sodium (mEq/L)	142 ± 4	142 ± 2	NS
Potassium (mEq/L)	4.8 ± 0.7	4.9 ± 0.5	NS
Corrected Ca (mg/dL)	9.1 ± 0.6	9.0 ± 0.6	NS
Phosphate (mg/dL)	5.0 ± 1.0	5.0 ± 1.4	NS
Total protein (g/dL)	6.8 ± 0.5	6.7 ± 0.6	NS
Albumin (g/dL)	3.8 ± 0.4	3.7 ± 0.5	NS
Intact PTH (pg/mL)	158 ± 120	164 ± 116	NS
Total cholesterol (mg/dL)	158 ± 35	161 ± 29	NS
LDL cholesterol (mg/dL)	88 ± 26	90 ± 25	NS
Triglycerides (mg/dL)	126 ± 67	115 ± 65	NS
Hs-CRP (mg/dL)	0.45 ± 0.86	0.56 ± 0.81	NS
β_2_-microglobulin (mg/L)	23.8 ± 6.2	24.2 ± 8.3	NS
Ferritin (ng/mL)	119 ± 116	112 ± 130	NS
TSAT (%)	34.5 ± 13.4	33.0 ± 9.8	NS
Total carnitine (μmol/L)	41.1 ± 10.8	38.1 ± 8.3	NS
Free carnitine (μmol/L)	26.7 ± 7.6	23.9 ± 6.3	NS
Acyl carnitine (μmol/L)	14.9 ± 4.8	14.3 ± 3.6	NS
Acyl/free carnitine	0.59 ± 0.25	0.62 ± 0.16	NS

BUN, blood urea nitrogen; Ca, calcium; Hs-CRP, high-sensitivity C-reactive protein; LDL, low-density lipoprotein, PTH, parathyroid hormone; TSAT, transferrin saturation; NS, not significant.

### 3.2. Effects of l-Carnitine Supplementation on Clinical Variables

There were no significant changes in most clinical variables, including serum electrolytes, iron, transferrin saturation, albumin, lipid profiles, and hs-CRP levels, within groups or between the control and l-carnitine group after 12 months (Table 2). There was, however, a significant ERI reduction in the l-carnitine group. Total, free, and acyl carnitine levels were significantly increased in the l-carnitine group after 12 months, whereas the acyl/free carnitine ratio was lower (Table 3). Total, free, and acyl carnitine levels in the l-carnitine group were significantly higher compared to the control group after 12 months of treatment.

**Table 2:** Effects of l-carnitine treatment on clinical variables.

	l-carnitine Group (*n* = 67)			Control Group (*n* = 64)		
	Baseline	12 Months	*p* Value	Baseline	12 Months	*p* Value
Hemoglobin (g/dL)	10.9 ± 0.9	11.0 ± 0.8	NS	10.9 ± 0.9	10.9 ± 1.0	NS
BUN (mg/dL)	55.2 ± 12.7	55.7 ± 14.5	NS	56.9 ± 12.7	57.8 ± 13.1	NS
Creatinine (mg/dL)	9.58 ± 2.52	9.48 ± 2.70	NS	9.49 ± 2.60	9.36 ± 2.40	NS
Uric acid (mg/dL)	7.0 ± 1.2	6.9 ± 1.1	NS	6.9 ± 1.0	6.9 ± 1.1	NS
Sodium (mEq/L)	142 ± 4	142 ± 4	NS	142 ± 4	142 ± 4	NS
Potassium (mEq/L)	4.7 ± 0.7	4.7 ± 0.7	NS	4.7 ± 0.6	4.8 ± 0.6	NS
Corrected Ca (mg/dL)	9.0 ± 0.6	9.0 ± 0.7	NS	8.9 ± 0.8	8.9 ± 0.7	NS
Phosphate (mg/dL)	5.1 ± 1.0	5.2 ± 1.3	NS	5.2 ± 1.1	5.1 ± 1.0	NS
Total protein (g/dL)	6.8 ± 0.5	6.7 ± 0.5	NS	6.7 ± 0.5	6.7 ± 0.5	NS
Albumin (g/dL)	3.8 ± 0.4	3.7 ± 0.4	NS	3.7 ± 0.4	3.7 ± 0.4	NS
AST (IU/L)	15 ± 9	15 ± 9	NS	14 ± 11	14 ± 9	NS
ALT (IU/L)	12 ± 8	12 ± 8	NS	11 ± 6	12 ± 8	NS
Total cholesterol (mg/dL)	160 ± 36	154 ± 31	NS	163 ± 34	160 ± 32	NS
LDL cholesterol (mg/dL)	89 ± 26	85 ± 22	NS	90 ± 26	88 ± 25	NS
Triglycerides (mg/dL)	127 ± 64	135 ± 74	NS	116 ± 64	118 ± 54	NS
Hs-CRP (mg/dL)	0.19 ± 0.20	0.16 ± 0.18	NS	0.21 ± 0.23	0.20 ± 0.20	NS
Ferritin (ng/mL)	123 ± 123	115 ± 103	NS	111 ± 70	104 ± 68	NS
TSAT (%)	30.5 ± 9.3	28.8 ± 10.2	NS	29.3 ± 11.0	29.4 ± 10.7	NS
β_2_-microglobulin (mg/L)	27.4 ± 8.7	26.6 ± 8.4	NS	27.6 ± 6.8	27.1 ± 5.7	NS
Intact PTH (pg/mL)	153 ± 88	150 ± 92	NS	147 ± 80	141 ± 82	NS
ERI	9.75 ± 9.02	6.39 ± 5.52	<0.01	10.7 ± 10.8	9.83 ± 10.4	NS

AST, aspartate aminotransferase; ALT, alanine aminotransferase; BUN, blood urea nitrogen; Ca, calcium; ERI, erythropoietin responsiveness index; Hs-CRP, high sensitivity C-reactive protein; LDL, low-density lipoprotein; PTH, parathyroid hormone; TSAT, transferrin saturation; NS, not significant.

**Table 3:** Changes in l-carnitine concentration during the treatment period.

	l-carnitine Group (*n* = 67)			Control Group (*n* = 64)		
	Baseline	12 Months	*p* Value	Baseline	12 Months	*p* Value
Total carnitine (μmol/L)	42.0 ± 11.0	243.1 ± 87.4 *	<0.0001	40.2 ± 9.7	49.9 ± 9.5	NS
Free carnitine (μmol/L)	27.0 ± 7.8	157.6 ± 54.8 *	<0.0001	25.9 ± 6.9	25.6 ± 6.7	NS
Acyl carnitine (μmol/L)	15.0 ± 5.0	85.6 ± 38.3 *	<0.0001	14.2 ± 4.5	14.3 ± 4.5	NS
Acyl/free ratio	0.59 ± 0.27	0.54 ± 0.17	<0.05	0.56 ± 0.17	0.58 ± 0.19	NS

* *p* < 0.0001 *vs.* control group. NS, not significant.

### 3.3. Effects of l-Carnitine Supplementation on baPWV

**Figure 2:**
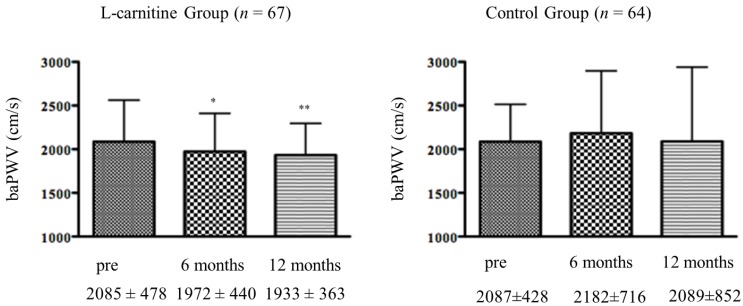
Changes in baPWV following l-carnitine treatment. baPWV, brachial-ankle pulse wave velocity; ^*^ *p* < 0.05, ^**^ *p* < 0.001 *vs.* pre value.

**Figure 3:**
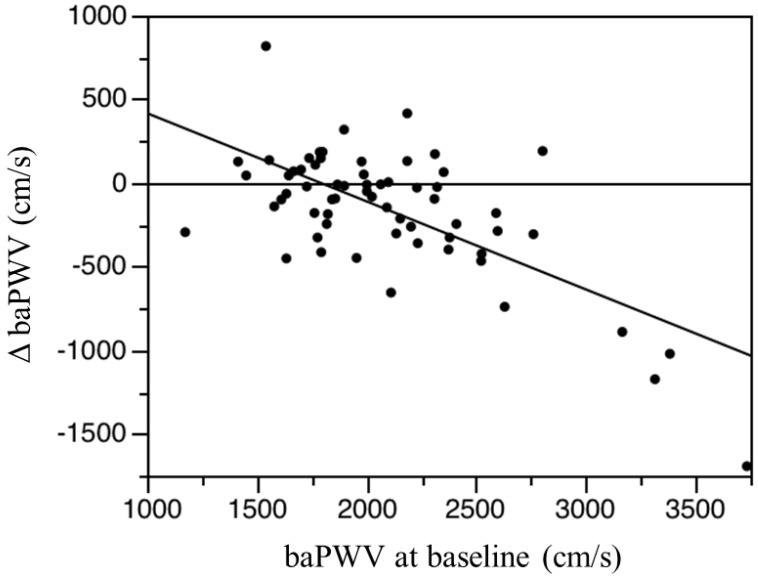
Linear regression analysis of the association between ΔbaPWV (differences between the 12-month baPWV and baseline baPWV) and baseline baPWV (brachial-ankle pulse wave velocity) in the l-carnitine treatment group. *Y*= −0.527*X* + 943.604, *r* = −0.677, *p* < 0.0001, *n* = 67.

As shown in Figure 2, the baPWV values at six months and 12 months were significantly lower than baseline in the l-carnitine group but not in the control group. Systolic, diastolic, and mean blood pressure values did not change significantly in either group over the 12-month study period (Table 4). There was no significant relationship between baseline baPWV and baseline total, free, or acyl carnitine in any of the subjects. There was no significant association between ΔbaPWV and any measured clinical parameter, including age, HD duration, change in total, free, or acyl carnitine levels, or change in ERI. However, there was a significant correlation between ΔbaPWV and the initial baPWV magnitude (*r* = −0.677, *p* < 0.0001; Figure 3). That is, greater baseline baPWV was associated with a larger baPWV decrease after 12 months of oral l-carnitine treatment. In multiple stepwise regression analysis, baseline baPWV was the sole independent predictor of ΔbaPWV (*R*^2^ = 0.856, *p* = 0.0012).

**Table 4:** Changes in blood pressure and heart rate during the treatment period.

	l-carnitine Group (*n* = 67)				Control Group (*n* = 64)			
	Baseline	12 Months	6 Months	*p* Value	Baseline	12 Months	6 Months	*p* Value
SBP (mmHg)	151 ± 25	149 ± 23	149 ± 18	NS	150 ± 18	151 ± 20	147 ± 22	NS
DBP (mmHg)	82 ± 14	81 ± 12	81 ± 11	NS	78 ± 13	81 ± 11	82 ± 14	NS
MAP (mmHg)	114 ± 19	114 ± 17	113 ± 14	NS	113 ± 16	114 ± 18	112 ± 16	NS
HR (bpm)	73 ± 12	73 ± 11	74 ± 11	NS	71 ± 10	74 ± 21	71 ± 14	NS

DBP, diastolic blood pressure; HR, heart rate; MAP, mean arterial pressure; SBP, systolic blood pressure; NS, not significant.

## 4. Discussion

The risk of death due to CVDs, such as ischemic heart disease, cerebrovascular diseases, and heart failure, is 10–30-fold greater in dialysis patients relative to the general population [20]. Compared to the general population, dialysis patients show a two-to-five-fold greater risk of incident acute myocardial infarction and poorer survival [21]. This is also true for cerebrovascular diseases [22], as the high incidence and fatality rate synergistically increase the risk of death due to CVD [23]. The prognosis of dialysis patients after lower limb amputation is also extremely poor [24,25], and the hospital death rate is high [22]. Dossa *et al.* reported that the hospital death rate after lower limb amputation was 7% in non-dialysis patients but 24% in dialysis patients, while the two-year survival rate after lower limb amputation was 79% in non-dialysis patients but only 27% in dialysis patients [24]. Aulivola *et al.* [25] also reported extremely poor prognosis for dialysis patients after lower limb amputation (particularly major amputation). Dialysis patients with peripheral artery disease (PAD) also exhibit high hospitalization rates [26], risk of death [27], death within six months after initiation of dialysis [28], and mortality rate after acute myocardial infarction [29]. The reported total risk of death is 7.09-fold higher in patients with an ABI of less than 0.9 and 2.20-fold higher in patients with an ABI of 1.3 or above compared to individuals with an ABI of 1.1–1.3. A high baPWV measured in the brachium and ankle is another prognostic factor in dialysis patients [30,31], as its value falsely decreases in patients with obstructive arteriosclerosis in the lower limbs. Therefore, caution is needed and simultaneous measurement of the ABI may be helpful. Although evaluation of these non-invasive surrogate indices may help estimate the individual CVD risk, the criteria used for their evaluation and the appropriate frequency of their use have not been established [32]. In the present study, we excluded patients with abnormal ABI. Although it has been reported that baPWV values are affected by blood pressure [33], there were no significant intra- or intergroup differences in blood pressure during the study period. Therefore, we suggest that l-carnitine therapy had an anti-atherosclerotic effect since baPWV values were significantly decreased in the l-carnitine group.

Oxidative stress in ESKD patients arises from reactive oxygen species (ROS) generated in activated endothelial cells [34]. These ROS alter vascular tone by reacting with vasodilatory NO and thereby increase the risk of CVD, the major cause of death for ESKD patients [34]. Carnitine administration increased prostaglandin I_2_ and NO production and decreased ROS production in animal models [35]. Furthermore, plasma levels and myocardial expression of IL-1β, IL-6, and TNF-α are decrease by l-carnitine treatment in Nω-nitro-l-arginine methyl ester-treated rats [36]. The antioxidant efficacy of l-carnitine has been reported in aging, atherosclerotic rats, hypercholesterolemic rabbits, and spontaneously hypertensive rats, all of which exhibit an increase in systemic oxidative stress [36]. Furthermore, it has been reported that l-carnitine treatment improves insulin resistance, increases protein synthesis, and has an anti-inflammatory effect in HD patients [37,38,39]. Therefore, the reduced baPWV in l-carnitine-treated patients in the present study may be due, at least in part, to antioxidant and/or anti-inflammatory effects, although further investigations are be needed to clarify the precise mechanisms.

Poor ESA responsiveness is linked to inflammation, malnutrition and, most importantly, to CVD, and called MIA syndrome or MICS is strongly associated with a greater risk of death in patients with ESKD [6,7,40,41]. l-carnitine administration improves the response to ESA in HD patients by increasing hemoglobin, decreasing the ESA dose required, or by decreasing ERI [42]. In the present study, ERI was also significantly reduced in the l-carnitine group. Furthermore, oxidative stress and reactive carbonyl compounds may contribute to the formation of advanced glycation end products (AGEs), which accumulate at an accelerated rate in diabetes and ESKD, thereby promoting the development and progression of CVD [43,44]. An *in vitro* study found that l-carnitine inhibits the AGE modification of bovine serum albumin, and this antiglycating capacity is more potent than that of aminoguanidine, a prototype inhibitor of AGE [45]. Furthermore, administration of l-carnitine significantly reduces skin AGE levels in patients with HD [46]. Low carnitine levels are also associated with increased risk of CVD in uremic patients [47]. Administration of l-carnitine attenuates the development and progression of atherosclerosis in an animal model [48] and improves endothelial dysfunction and ameliorated cardiac dysfunction in patients with HD [47]. Thus, carnitine deficiency may contribute to CVD by promoting inflammation and oxidative stress.

In the present study, a lower baPWV was observed in 43 of 67 subjects (67%) after 12 months of l-carnitine therapy, and the decrease was generally largest in patients with higher basal baPWV as revealed by linear regression (Figure 3). We monitored baPWV as a marker of atherosclerosis, but could not assess actual vascular calcification using computed tomography or ultrasonography. Therefore, whether l-carnitine supplementation prevents the progression of atherosclerosis requires future serial imaging studies in HD patients. Furthermore, long-term investigations are necessary to accurately assess the preventive efficacy of l-carnitine supplementation on CVD risk in HD patients.

## 5. Conclusions

In conclusion, the present study demonstrates that l-carnitine supplementation significantly decreases baPWV, a marker for atherosclerosis, in HD patients with carnitine deficiency. These observations suggest that l-carnitine may be a viable therapeutic strategy for preventing atherosclerotic CVD.
